# Protocol for the preparation of flexible material from the mycelium of wood-decay fungal strains and mechanical property investigation

**DOI:** 10.1016/j.xpro.2026.104391

**Published:** 2026-02-27

**Authors:** Dhanalakshmi Vadivel, Daniele Dondi

**Affiliations:** 1 Laboratory of Radiation and EPR Spectroscopy, Department of General Chemistry, University of Pavia, Via Torquato Taramelli 12, 27100 Pavia, Italy

**Keywords:** Chemistry, Material sciences, Environmental sciences

## Abstract

Extraction of chitin-glucan-based materials from fungi has several advantages over animal-based chitin. Here, we present a protocol for extracting chitin-glucan material from the mycelium of wood-decay fungal strains and investigating its mechanical properties. We describe steps for extracting chitin-glucan-based material, using acetic acid as a crosslinking agent to improve mechanical properties, and adding glycerol to increase flexibility. We then detail procedures for investigating tear strength and elongation at break, as well as establishing the required properties of flexible materials.

For complete details on the use and execution of this protocol, please refer to Vadivel et al.^1^

## Highlights

• A step-by-step guide to develop flexible bio-based materials
• Improved mechanical properties by acetic acid and plasticizing with glycerol
• Details to characterize Young’s modulus, tear strength, and elongation at break

## Abstract (editor highlights)

Publisher’s note: Undertaking any experimental protocol requires adherence to local institutional guidelines for laboratory safety and ethics.

## Graphical abstract

**Figure:**
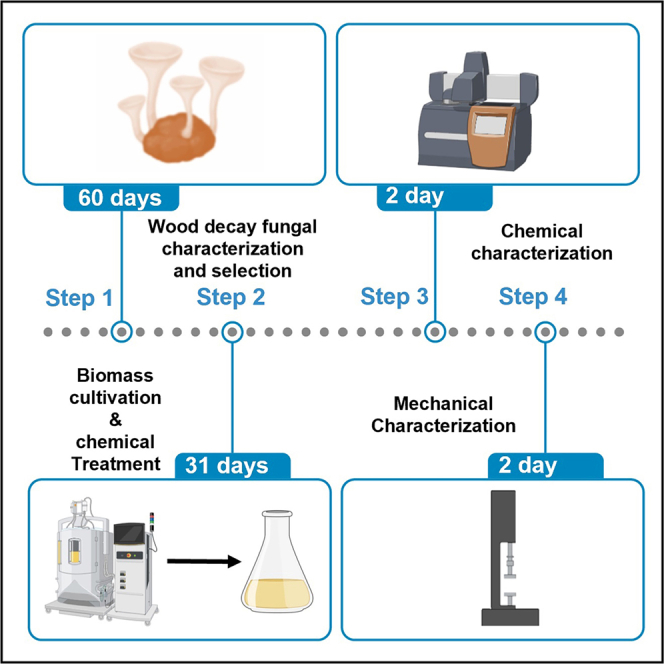

## Before you begin

Owing to their flexibility, the preparation of elastomeric materials that are leather alternatives can completely remove the environmental and ethical (animal hides) concerns when prepared from fungal sources.^2^ The environmental concerns are still real for synthetic alternatives, such as polyurethanes and polyvinyl chloride.^2^ Chitin, a polysaccharide, is an excellent leather substitute and is primarily extracted from shrimp, crab and lobster shells as support structures.^1^^,^^3^ Alternatively, chitin extraction from fungi is advantageous due to no impacts on animals, absence of allergens, absence of harsh extractions, alterability in properties of fungal-derived chitins, and year-round harvestability of mushrooms (fungi).^1^

This protocol outlines the simple extraction procedure from the mycelium of wood decay fungal strains, the strengthening procedure for effective crosslinking and plasticising, and the necessary experiments for studying the mechanical properties of the prepared flexible sheet material.

### Innovation

The innovation lies in bridging biopolymer extraction with functional material engineering, demonstrating how fungal chitin-glucan can be transformed into flexible, durable materials. Compared with conventional methods that rely on animal sources or intensive chemical treatments, this approach is simpler, more eco-friendly, and better aligned with circular bioeconomy principles that offer a renewable alternative to animal-derived chitin. By incorporating acetic acid crosslinking to augment mechanical strength and glycerol plasticization to increase flexibility, the protocol produces biopolymer films with customized mechanical characteristics. Compared to traditional techniques, this method is more direct, environmentally sustainable, and consistent with the principles of a circular bioeconomy. This work presents an effective protocol for the extraction of chitin-glucan from the mycelium of wood-decaying fungi and the customization of its mechanical properties for flexible material applications.

## Key resources table

**Table:** 

REAGENT or RESOURCE	SOURCE	IDENTIFIER
**Chemicals, peptides, and recombinant proteins**		

Malt extract agar	Biokar Diagnostic	Product code: A1101
Dextrose	Sigma-Aldrich	CAS Number: 50-99-7
Yeast extract	Biokar Diagnostic	Product code: BK153HA
1% Acetic acid	Thermo Fisher Scientific	Catalogue number: 035569.AP
Glycerol	Sigma-Aldrich	Catalogue number: G7757-1L
Sodium Hydroxide	Sigma-Aldrich	Catalogue number: 567530-250GM

**Experimental models: Organisms/strains**		

Fungi	Herbarium Universitatis Ticinensis (DSTA, University of Pavia, Italy)	https://terraeambiente.dip.unipv.it/it/dipartimento/risorse-musei-e-laboratori/erbario-herbarium-universitatis-ticinensis-pav

**Software**		

STARe (TGA)	Mettler Toledo	https://www.mt.com/it/it/home/products/Laboratory_Analytics_Browse/TA_Family_Browse/TA_software_browse.html
Test works Version 4	Test works	N/A
TRIOS (DSC)	TA systems	https://www.tainstruments.com/support/software-downloads-support/downloads/#tab-1478023717256tbl1478023717256-9-7
MTS Insight Electromechanical Testing Systems 10 kN	(MTS System Corporation)	N/A
Mettler Toledo TGA1 XP1	Mettler Toledo	Catalog. NO:01-915-192
DSC 2010	TA systems	N/A
TA 2980 Dynamic-mechanical analyser	TA instruments	N/A
AR2000ex Rheometer	TA instruments	N/A

## Step-by-step method details

### Sample preparation

**Timing: 60 days (for step 1)**

**Timing: 18 days (for step 2a)**

**Timing: 8 days (for step 2b)**

**Timing: 8 days (for step 2c)**

**Timing: 1–2 days (for step 3)**

**Timing: 3 days (for step 4)**

**Timing: 1 day (for step 5)**

**Timing: 1 day (for step 6)**

**Timing: 1 day (for step 7)**

1. Selection criteria for fungal strains.^4^
  a. Determine chitin and glucan content on the cell wall of the fungi using thermogravimetric analysis (TGA) and scanning electron microscope (SEM).^5^

**CRITICAL:** Select the material with high chitin, ⍺-glucan, and β-glucan.

2. Fungal biomass cultivation which shown in Table 1.
  a. Fungal strains culture.
    i. Grow the fungal strain cultures in 2% malt extract agar on Petri plates of 9 cm diameter.
    ii. Grow the fungal strains for 10 days.
    iii. Draw Mycelium plugs of 0.5 cm^2^ from actively growing colonies.
    iv. Transfer the drawn mycelium plugs into the 0.1 L sterilised liquid broth of malt (2%), dextrose (0.5%), and yeast extract (0.2%).
    v. Keep the mycelium plugs and broth in a shaker incubator at 181 rpm at 25 °C in the dark for 7 days.
    vi. Remove and blend the mixture after 7 days using a sterile metal blender.
      **CRITICAL:** Use of sterilised materials and instruments is really important to avoid cross-contamination and aid in obtaining a good yield.
  b. Cultivation of fungal strain in a flask.
    i. Transfer the blended contents to a larger flask containing 0.5 L sterilised liquid broth of malt (2%), dextrose (0.5%), and yeast extract (0.2%).
    ii. Store the contents for three days.
    iii. Remove and blend the contents after three days using a sterile metal blender.
    iv. Separate 33 mL from the blended creamy mixture and add to the flask containing 1.2 L of sterilised liquid broth of malt (2%), dextrose (0.5%), and yeast extract (0.2%).
    v. Keep the flask in a shake incubator for 7 days at 181 rpm at 25 °C.
      **CRITICAL:** Following the same procedure.
  c. Cultivation of fungal strain in bioreactor.
    i. Transfer the blended contents to a larger flask containing 1 L sterilised liquid broth of malt (2%), dextrose (0.5%), and yeast extract (0.2%).
    ii. Store the contents for three days.
    iii. After three days, remove the contents and blend using a sterile metal blender.
    iv. Transfer the blended mixture into a 10 L bioreactor containing 10 L sterilised liquid broth of malt (2%), dextrose (0.5%), and yeast extract (0.2%).
    v. Add 2 mL of sunflower oil.
    vi. Set the bioreactor at 25 °C, 50% oxygen saturation, with changing aeration of 5-20 L/min, 80-150 rpm agitation and proceed for 7 days.
      **CRITICAL:** Addition of sunflower oil to the mixture limits the foam formation.

**Table 1:** Data comparison between fungal biomass production for the two different procedures

Strain^1^	Cultivation	Biomass production
		Dry biomass (g)
Abortiporus biennis (Bull.) Singer	Bioreactor	73 ±1
	Flasks	38 ±0.5
Fomitopsis pinicola (Sw.) P. Karst.	Bioreactor	37 ±0.7
	Flasks	31 ±0.6
Fomitopsis iberica Melo & Ryvarden	Bioreactor	51 ±0.3
	Flasks	44 ±0.5
Stereum hirsutum (Willd.) Pers.	Bioreactor	111 ±1.2
	Flasks	60 ±0.4
Coriolopsis gallica (Fr.) Ryvarden	Bioreactor	24 ±0.8
	Flasks	25 ±0.2

3. Extraction of alkali-insolubles.
  a. Wash the biomass obtained after cultivation with water; this removes the residues of the medium and extracellular compounds produced by the fungus.
  b. Filter the contents through a nylon filtration bag.
  c. Blend the biomass using a sterile metal blender.
  d. Keep the blended biomass in hot water at 90 °C for 1 hour, which removes water-soluble compounds.
  e. After partially cooling down, filter the biomass using nylon filtration bags.
  f. Wash and filter the biomass several times.
  g. Resuspend the recovered biomass in 1.5 L of water.
  h. Blend the mixture until it is homogeneous.
  i. Add 3.5% w/w of Sodium hydroxide (NaOH) to the solution.
  j. Incubate the mixture for 2 hours at 65 °C. This procedure is followed to eliminate proteins and alkali-soluble carbohydrates.
  k. Allow the mixture to cool to ambient temperature.
  l. Filter the mixture with a nylon filtration bag.
  m. Wash the biomass multiple times to reduce the pH to neutral.
  n. Drain the biomass completely and recover.
  o. Resuspend the biomass in water to form an alkali-insoluble slurry.

**CRITICAL:** Drain the contents completely during the last cycle of washing, it is necessary for maximising the recovery of biomass from the filtration bag.

4. Crosslinking and Plasticization of biomass.
  a. Add 1% acetic acid and 10% glycerol to a 3L final volume to the alkali-insoluble slurry.
  b. Blend the mixture to avoid coagulation and promote homogeneity.
  c. Incubate the obtained creamy mixture in a water bath at 60 °C for 4 hours.
  d. Pour the slurry on a filter paper sheet kept on the vacuum table of size (21 cm x 29.7 cm).
  e. Transfer the solid part to plastic foil (34.5 cm x 34.5 cm) and dry for 48 hours.

**CRITICAL:** It is important to wait till complete drying of the slurry using vacuum, as the slurry is thick and might not lose water.

***Note:*** During the sodium hydroxide treatment step, a partial deacetylation of chitin can occur, leading to free amine groups. The addition of acetic acid protonates amine groups, creating positively charged groups that increase the chain interaction through dipole-induced dipole forces, which enhances the stability of the mycelium mats, rather than being brittle in the presence of chitin. In this case, we can not expect the acetic acid to readily dissolve the material^6^^,^^7^ because the naturally packed structure of the polymers does not allow it. The introduction of glycerol improves the flexibility of the mycelium mats through the formation of hydrogen bonds with chitin and beta-glucan backbones, which significantly affects the mechanical properties.^8^ The right tuning with the acetic acid that brings stability and glycerol that brings flexibility is the key.

**Figure 1:**
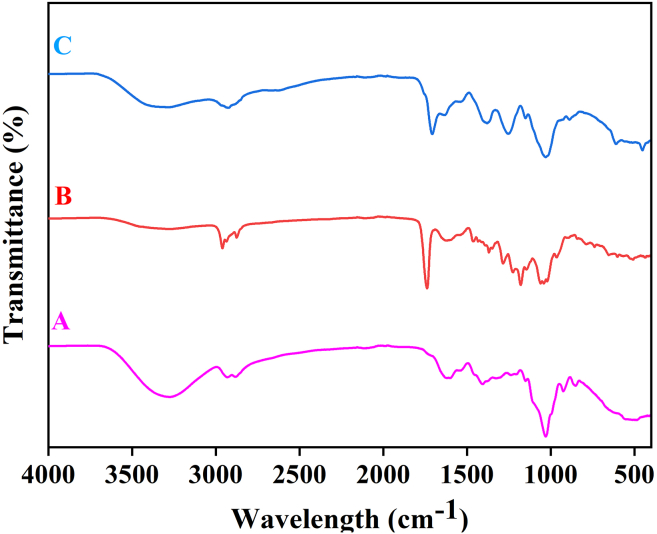
FTIR of mycelium mat with glycerol, acetic acid, and both FTIR of mycelium mat with glycerol alone (A), FTIR of mycelium mat with acetic acid alone (B), FTIR of mycelium mat with both glycerol and acetic acid (C).

Figure 1A shows the mycelium mat containing only glycerol plasticizer, the sharp peak around 1028 cm^-1^ corresponds to C-O bond stretching of secondary alcohol in glycerol which is also a characteristic peak.^9^ Figure 1B shows the mycelium mat containing only acetic acid as crosslinking agent, the sharp peak around 1748 cm^-1^ represents the C=O stretching frequency which is a characteristic peak in acetic acid.^10^ Figure 1C shows the mycelium mats containing both characteristic peaks of acetic acid and glycerol shows preliminary crosslinking and plasticization effect which could be confirmed further by mechanical properties experiments.

5. Thermogravimetric Analysis.
  a. Set the conditions in TGA as 25 °C to 800 °C by the heating rate of 20 °C/min under Nitrogen atmospheric conditions with a mass flow rate of 100 mL/min.
  b. Perform a Blank with an empty crucible.
  c. Add 8-10 mg of sample and run the experiment.
  d. Analyse the data as follows,
    i. Complete the experiment and analyse the data using the StarE software.
    ii. Take the first derivative of the analysed sample.
    iii. Plot the data as a function of % of weight loss vs temperature.
6. Mechanical testing system.
  a. Cut rectangular specimens (110 mm × 10 mm) specimen of the formed sheets.
  b. Set pneumatic grips 50 mm apart from each other.
  c. Use 250 N load cells.
  d. Set the separation of jaws at a uniform speed of 2 mm/min, this was to ensure quasi-static conditions.
  e. Measure each sample with a digital calliper and take the weight of the sample before mechanical testing to estimate its density.
  f. Test the samples and analyse the data as follows,
    i. Calculate the tensile strength *Tn* (in MPa) $Tn=\frac{F}{wt}$ where *F* is the highest force recorded during the test (in N), *w* is the specimen width (in mm), and *t* is the specimen thickness (in mm).
    ii. Calculate the percentage elongation εp to a specified load value, with εp = $\frac{L-L_{0}}{L_{0}}$ 100 where *L* is the separation of the jaws and *L*_*0*_ is the initial separation of the jaws.
7. Dynamic mechanical analysis.
  a. Produce the specimen with 0.8 mm thickness.
  b. Set the frequency as 1 Hz.
  c. Measure Storage (E′, G′) and loss (E″, G″) moduli as a function of temperature in the 20°C/200°C range.

## Expected outcomes

**Figure 2:**
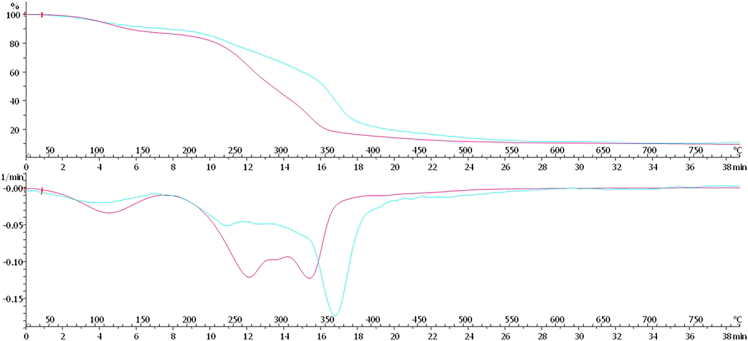
General representation of TGA curves with the first derivative Figure reprinted with permission from Vadivel et al., 2024.^1^

**Figure 3:**
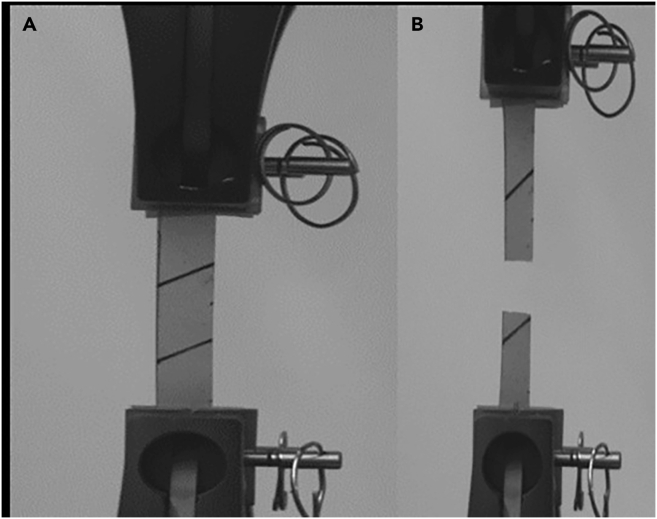
Mechanical testing on produced myco-sheets General representation of the mechanical testing applied to formed myco-sheets, (A) Clamped sample before and (B) after the test.^1^ Figure reprinted with permission from Vadivel et al., 2024.

**Figure 4:**
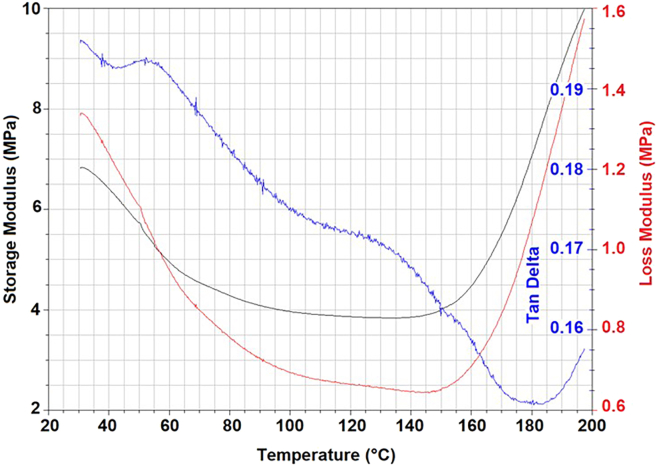
General representation of Dynamic mechanical analysis for Elastomeric material Figure reprinted with permission from Vadivel et al., 2024.^1^

**Figure 5:**
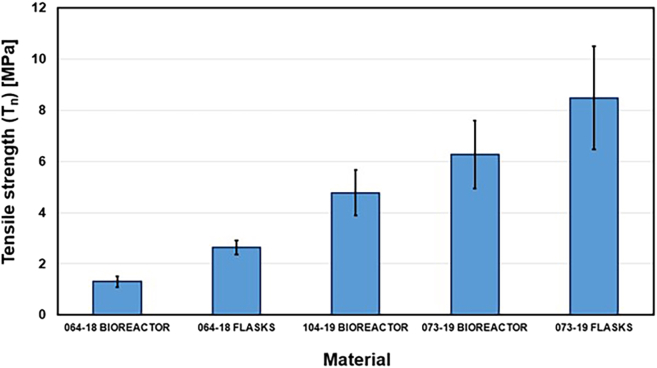
Graphical comparison of average tensile strength of prepared chitin-glucan myco-materials Figure reprinted with permission from Vadivel et al., 2024.^1^

**Figure 6:**
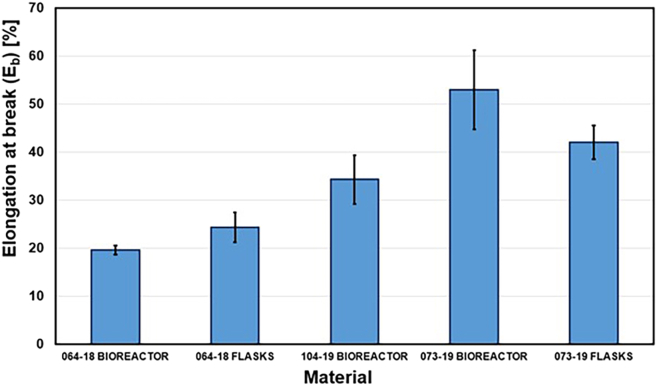
Graphical comparison of average elongation at break of prepared chitin-glucan myco-materials Figure reprinted with permission from Vadivel et al., 2024.^1^

**Figure 7:**
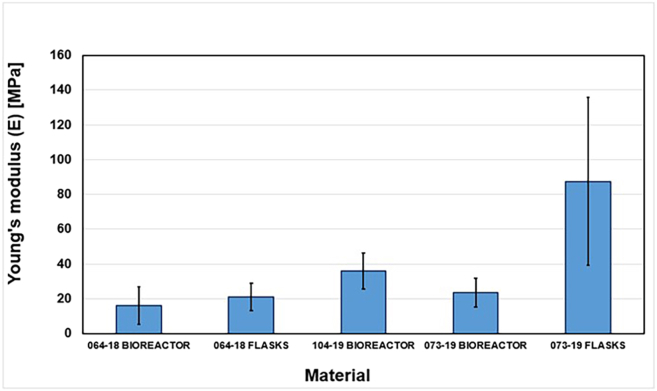
Graphical comparison of average Young’s modulus of prepared chitin-glucan myco-materials Figure reprinted with permission from Vadivel et al., 2024.^1^

The study provides a detailed examination of methods for obtaining mycelium from wood-decaying fungi, ensuring a reliable source of biomass for biopolymer extraction. A step-by-step procedure is outlined for cultivating fungal biomass and subsequently extracting chitin–glucan material, enabling reproducibility and clarity in the preparation process. To establish the performance of the resulting biopolymer, comprehensive chemical and mechanical characterizations are conducted, including analyses of structural composition and tests of mechanical strength (Figures 2, 3, 4, 5, 6, and 7). These investigations not only confirm the stability of the material but also elucidate the underlying reasons for its durability and flexibility, thereby offering valuable insights into its potential applications as a sustainable alternative to conventional materials.

## Limitations

The main limitation of this protocol resides in the selection of strain, the process of selecting the strain, finding an appropriate cultivation method, and ensuring quantitative extraction of material, which poses immense challenges with the requirement of huge resources. During the treatment of biomass obtained from liquid culture, if the particle size is lower than the pore size of the filter, it is highly difficult to achieve good filtration and washing to extract chitin-glucan material for the production of myco-sheets.

## Troubleshooting

### Problem 1

Avoiding the formation of cracks in the curved parts of the specimen while cutting in a Dog-bone-like structure.

### Potential solution

Cut the specimen in a rectangular fashion.

### Problem 2

For a better understanding of Thermogravimetric measurements to better estimate the temperature of the decomposition peaks and to locate the start/end of the decomposition.

### Potential solution

Use the first derivative of the analysed data for better understanding.

### Problem 3

Preparing materials with a higher elastic nature is difficult.

### Potential solution

In addition, commercially available plasticisers or glycerol can enhance the elasticity of the material.

### Problem 4

Selecting the mode of cultivation of fungi between bioreactor and flask cultivation.

### Potential solution

Bioreactor cultivation of fungi had merits as the materials showed more elastic properties compared to flask-cultivated fungi, as the monitoring of pH, dissolved oxygen in the growing medium, aeration and agitation is possible with bioreactors.

## Resource availability

### Lead contact

Further information and requests for resources and reagents should be directed to and will be fulfilled by the lead contact, Dhanalakshmi Vadivel (dhanalakshmi.vadivel@unipv.it).

### Technical contact

Questions about the technical specifics of performing the protocol should be directed to the technical contact, Daniele Dondi (daniele.dondi@unipv.it).

### Materials availability

This study did not generate new unique reagents.

### Data and code availability

The published protocol contains all of the data/code generated during this study. The datasets used and/or analyzed during this study are available from the corresponding author on reasonable request.

## Acknowledgments

D.D. and D.V. acknowledge support from the Ministry of University and Research (MUR) and the 10.13039/501100004769University of Pavia through the program “10.13039/100017336Dipartimenti di Eccellenza 2023–2027.” The graphical abstract/figures were created using Biorender.com.

This project has been funded by 10.13039/501100002803Fondazione Cariplo, grant no. 2018-1765, entitled “Myco-advanced leather materials (MATER).”

## Author contributions

D.V. designed the study, discussed the data, and wrote the manuscript. D.D. designed the study and discussed the data.

## Declaration of interests

The authors declare no competing interests.
